# Potential protective effects of red grape seed extract in a rat model of malathion-induced neurotoxicity

**DOI:** 10.14202/vetworld.2023.380-385

**Published:** 2023-02-26

**Authors:** Mohamed Jamal Saadh

**Affiliations:** 1 Department of Basic Science, Faculty of Pharmacy, Middle East University, Amman, Jordan;; 2 Applied Science Research Center, Applied Science Private University, Amman, Jordan

**Keywords:** apoptosis, malathion toxicity, oxidative stress, pesticide, red grape seed extract

## Abstract

**Background and Aim:**

Exposure to pesticide mixtures used in agricultural practice poses a grave risk to non-target animals. This study aimed to determine whether red grape seed extract (RGSE, which is 95% bioflavonoids and equal to 12,000 mg of fresh red grape seed, and 150 mg of vitamin C) alleviated the changes in brain-derived neurotrophic factor (BDNF) level, acetylcholinesterase activity, oxidative stress, and apoptosis induced by orally administered malathion in a rat model of malathion-induced neurotoxicity.

**Materials and Methods:**

Thirty-two adult male Wistar albino rats were divided into four groups and exposed to malathion with or without 4 weeks of RGSE treatment, treated with RGSE alone, or left untreated as controls. The animals were euthanized 24 h after last treatment. Brain samples were collected to measure acetylcholinesterase, superoxide dismutase (SOD), and caspase 3 activity, total antioxidant capacity (TAC), and BDNF levels.

**Results:**

Malathion significantly reduced acetylcholinesterase and SOD activity and TAC and significantly increased caspase 3 activity. In comparison, acetylcholinesterase and SOC activity, BDNF level, and TAC were improved and caspase 3 activity was decreased in the malathion-RGSE group, indicating that RGSE corrected the alterations detected in these biochemical parameters.

**Conclusion:**

Oxidative stress and apoptosis in the brains of rats exposed to oral malathion were substantially controlled by RGSE treatment.

## Introduction

Malathion is one of the most widely used organophosphorus pesticides used to eliminate house bugs, flies, mosquitoes, and human lice [1]. Its intended application as well as the high risk of food contamination following its use might lead to severe environmental and workplace contamination [2]. Immediate toxic effects of malathion are mostly due to the inhibition of acetylcholinesterase activity in the central nervous system, which leads to higher levels of acetylcholine and a variety of cholinergic symptoms [3].

Several studies have suggested that organophosphorus pesticides might alter other biological targets as well. Brocardo *et al*. [4] reported that malathion poisoning impaired antioxidant response and increased the levels of several oxidative stress indicators in a dose-dependent manner. Moreover, previous studies have demonstrated malathion-induced brain injury and caspase 3 dependent neurotoxicity in rats [5, 6]. Importantly, organophosphorus pesticide intoxication may cause neuropsychiatric symptoms such as anxiety and depression, which can disrupt neurogenesis [7–9].

As the most active neurotrophic factor in the central nervous system, brain-derived neurotrophic factor (BDNF) supports neuronal health and survival [10] through many aspects, including neurogenesis, dendritic branching, neurotransmitter generation and release, synapse formation and maturation, and synaptic plasticity [11]. These biological effects of BDNF are mediated through its interaction with the receptor tyrosine kinase B [12].

Grape seed extract (GSE) is a popular nutritional supplement and a cheap source of antioxidants. The phenolic compounds in GSE include phenolic acids and related compounds as well as flavonoids [13]. Proanthocyanidins in GSE are considered to protect against myocardial ischemia/reperfusion injury and cardiomyocyte apoptosis caused by reactive oxygen species. In addition, GSE was shown to be beneficial in a variety of disorders, including skin aging and to exhibit anti-inflammatory, antiapoptotic, antinecrotic, cardiovascular, and anticancer effects [13]. Grape seed extract was also demonstrated to alleviate brain damage in a model of forebrain ischemia in adult gerbils [14]. In newborn rats, GSE can reduce brain damage and alleviate hypoxia-induced lipid peroxidation in brain [15].

Red grape seed extract (RGSE) and vitamin C is a rich source of plant-based antioxidants, which are crucial in defense against oxidative stress and free radical damage. This study aimed to determine whether RGSE altered BDNF levels, cholinergic activity, oxidative stress, and apoptosis in a rat model of neurotoxicity caused by oral malathion administration.

## Materials and Methods

### Ethical approval

All animal experiments were performed in accordance with the guidelines of the National Council for Animal Experimentation Control, and the Ethical Committee approval was obtained from Ethical Committee of Middle East University-Jordan (approval no. 2022MEU02).

### Study period and location

This study was conducted from January 2022 to June 2022 in Middle East University, Amman, Jordan.

### Materials

Malathion (purity 98.9%) was purchased from Sigma-Aldrich (Saint Louis, MO, USA). Red Grape seed extract was purchased from NZ Pure Health (Auckland, New Zealand) (each NZ Pure Health RGES capsule provides 100 mg of RGSE, which is 95% bioflavonoids and equal to 12,000 mg of fresh red grape seed, and 150 mg of vitamin C).

### Animals

Thirty-two adult male Wistar albino rats, aged 60 days and weighing between 260 and 280 g, were acquired from the Middle East University Pharmacy Faculty in Amman, Jordan. Rats were provided regular rodent food for 1 week before experiments for acclimation to the laboratory environment. Rats were housed in a group of four in cages, fed a standard meal, given access to unlimited water, and housed at a temperature of 23°C–25°C. In male Wistar albino rats, malathion dose required for 50% mortality (L_D5_0) is 1768–1857 mg/kg body weight [16]. In this study, malathion was used at a dose of 1132.5 mg/kg body weight, which was equivalent to 0.6 LD_50_. Briefly, malathion was dissolved in corn oil to prepare a suspension, and a customized syringe and needle with a ball tip was used for oral malathion administration. Laboratory animal care and handling practices were carried out in accordance with ethical standards for laboratory animals.

### Experimental design

Animals were split into the following four groups, with eight rats/group: Control group, including animals administered 1 mL/day corn oil once; malathion group, including animals administered 1 mL malathion-containing corn oil once; RGSE group, including animals administered 250 μg/kg RGSE once daily for 4 weeks, and malathion-RGSE group including animals administered 1 mL malathion-containing corn oil once before treatment with 250 μg/kg RGSE once daily for 4 weeks.

At the end of experiments, all animals were euthanized by exsanguination 24 h after last treatment. Whole brain samples from all groups were collected, and 1 g brain tissue per animal was homogenized in 10 mL phosphate buffer (pH 7.4). The homogenates were centrifuged at 3000× *g* for 5 min. Collected supernatants were stored at −20°C until biochemical analyses.

### Biochemical assay

Acetylcholinesterase activity was measured using an acetylcholinesterase activity assay kit (Sigma-Aldrich, St. Louis, USA) based on Ellman’s method. Total antioxidant capacity (TAC) was measured using a complete antioxidant capacity test kit (Bio Diagnostic Co. in Cairo, Egypt) based on the method described by Koracevic *et al*. [17]. Brain-derived neurotrophic factor levels were measured using a Human BDNF ELISA kit from Boster Biological Technology (Encyclopedia Cir., Fermont, CA), according to the manufacturer’s instructions. Brain BDNF levels were expressed as pg/g of tissue [18]. Activity levels of the endogenous antioxidant enzyme superoxide dismutase (SOD) were measured using SOD assay kit (Caymanv Chemicals Company, Ann Arbor, MI, USA) following the manufacturer’s instructions. Superoxide dismutase activity was expressed as U/mg of protein. Caspase 3 activity was measured using the Caspase-3 Colorimetric Activity Assay Kit (Chemicon, Temecula, USA) [19].

### Statistical analysis

GraphPad Prism 5.0 (https://graphpad-prism.software.informer.com/5.0/) and Statistical Package for the Social Sciences v.5.0 (IBM Corp., NY, USA) were used for statistical analyses. Differences among study groups were determined using a one-way analysis of variance followed by *post hoc* Tukey’s multiple comparisons test. p < 0.05 was considered statistically significant for all analyses.

## Results

**Figure-1:**
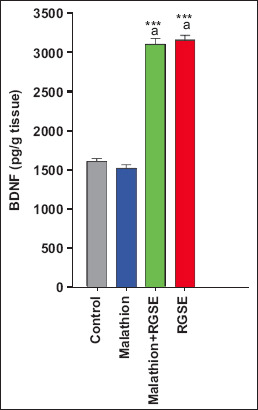
The level of BDNF as pd/g (mean ± SE) in rat brain tissue for all studied groups. ***p < 0.0001 (malathion or RGES or malathion + RGES compare with control group). ^a^p < 0.0001 (RGES or malathion + RGES compare with malathion group). BDNF=Brain-derived neurotrophic factor, SE=Standard error, RGSE=Red grape seed extract.

Brain acetylcholinesterase activity was significantly lower in the malathion group than in the control, RGSE, and malathion-RGSE group. Compared with the level of BDNF (1601 ± 37 pg/g tissue), caspase 3 activity (6 ± 0.1 U/g tissue), acetylcholinesterase activity (4.3 ± 0.23 U/mg tissue), TAC (51 ± 0.6 mM/g tissue), and SOD activity (0.267 ± 0.013 U/mg tissue) in control group, malathion administration led to increase in BDNF level (1516 ± 42.5 pg/g tissue, p > 0.05) and caspase 3 activity (10.3 ± 0.23 U/g tissue, p < 0.0001) and decreases in BDNF level (1516 ± 42.5 pg/g,), acetylcholinesterase activity (3.03 ± 0.27 U/mg tissue, p = 0.003), TAC (28.6 ± 2 mM/g tissue, p < 0.0001), and SOD activity (0.123 ± 0.007 U/mg tissue, p < 0.0001) (Figure-1).

**Figure-2:**
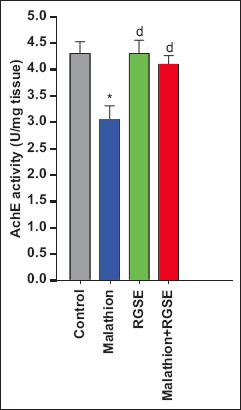
The activity of AchE is represented as U/mg (mean ± SE) in rat brain tissue for each group under study. *p < 0.05 (Malathion group compare with control group). ^d^p < 0.001 (RGES or malathion + RGES group compare with control group). AchE=Acetylcholinesterase, SE=Standard error, RGSE=Red grape seed extract.

**Figure-3:**
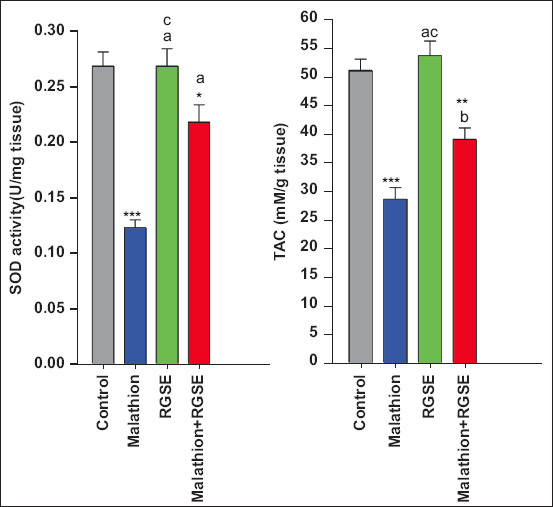
TAC as nM/g (mean ± SE), SOD U/mg (mean ± SE) in rat brain tissue for all studied groups. *p < 0.05, **p < 0.001, ***p < 0.0001, cNon significant correlation (malathion or RGES or malathion + RGES group compare with control group). ^a^p < 0.0001, ^b^p < 0.01, (RGES or malathion + RGES compare with malathion group). TAC=Total antioxidant capacity, SE=Standard error, SOD=Superoxide dismutase, RGSE=Red grape seed extract.

Total antioxidant capacity, SOD activity, and caspase 3 activity were slightly, albeit not significantly, higher in the RGSE group than in the control group (53.6 ± 2.39 mM/g tissue, 2.675 ± 0.155 U/mg protein, and 6.4 ± 0.0.18 U/g tissue, respectively). In contrast, brain BDNF level was significantly higher in the RGSE group than in the control group (3092 ± 80 pg/g tissue, p < 0.0001) (Figures-1–3).

**Figure-4:**
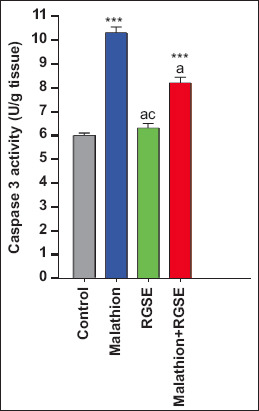
Caspase 3 activity U/g (mean ± SE) in rat brain tissue for all studied groups. ***p < 0.0001, ^c^Non significant correlation (malathion or RGES or malathion + RGES group compare with control group). ^a^p < 0.0001, ^c^Non significant correlation (RGES or malathion + RGES group compare with malathion group). SE=Standard error, RGSE=Red grape seed extract.

Finally, acetylcholinesterase activity, BDNF level, TAC, and SOD activity were significantly higher in the malathion-RGSE group than in the malathion group (p = 0.0018, p < 0.0001, and p = 0.003, respectively, Figures-1 and 2) whereas caspase 3 activity was significantly lower in the malathion-RGSE group than in the malathion group (p < 0.0001) (Figure-4).

## Discussion

As the most abundant and widely expressed trophic factor in the developing and mature mammalian brain [20], BDNF supports neuronal survival by regulating their growth, maturation (differentiation), and maintenance [20]. In this study, we found that malathion did not significantly increase BDNF protein levels in the rat brain. Dorri *et al*. [21] reported that malathion (50 mg/kg/day, IP for 2 weeks) significantly reduced BDNF levels (p < 0.01) in rat hippocampi.

In this study, oral administration of 250 mg/kg RGSE daily for 4 weeks resulted in a significant increase in brain BDNF levels in the RGSE and malathion-RGSE groups compared to the malathion group. *In vivo* administration of purified flavonoids has been shown to phosphorylate cAMP response element-binding protein in the hippocampus, leading to increases in the extracellular signal-regulated protein kinase and BDNF levels [22–24]. Some polyphenols act like neurotrophins by acting as direct tyrosine kinase B receptor agonists, while others turn on pathways that have neurotrophic effects. [24, 25]. Studies have demonstrated that specific flavonoids in RGSE correlate with its antioxidant effect. Grape flavonoids have a variety of biological effects, such as scavenging of free radicals, prevention of lipid oxidation, and reduction of hydrogen peroxide production [26].

In this study, the significant reduction in TAC detected in malathion-administered rats was significantly improved following RGSE treatment after malathion administration. Organophosphorus pesticide toxicity might induce oxidative stress due to redox cycling activity and free radical production [27]. These results imply that reactive oxygen species might be responsible for brain damage and altered behavior induced by malathion exposure [5]. Organophosphorus pesticides have been reported to alter Ca^+2^ homeostasis [28], leading to mitochondrial dysfunction possibly due to altered respiratory chain enzyme activity [29], and precipitated by the high energy demand of the brain and its poor antioxidant system[30].

In the current study, acetylcholinesterase level was significantly higher in the malathion-RGSE group than in the malathion group. Acetylcholinesterase inhibition cannot fully account for the wide variety of adverse effects associated with malathion exposure. The harmful consequences of acute and chronic malathion exposure have been largely attributed to oxidative stress. As a highly lipophilic compound, malathion immediately interacts with the phospholipid bilayer of the cellular membrane, leading to structural instability, and free radical generation [31].

In this study, TAC and SOD activities were significantly higher in the malathion-RGSE group than in the RGSE. Previous studies have demonstrated that malathion disrupts antioxidant balance by reducing the activity of cellular antioxidant mechanisms involving SOD, reduced glutathione, and glutathione peroxidase [31–35]. Other study has shown that malathion leads to increased antioxidant enzyme activity as an additional defense mechanism [36].

Brain is susceptible to severe oxidative damage because its high lipid composition renders the brain susceptible to oxidation and generation of reactive oxygen species due to high oxygen consumption in the presence of relatively low antioxidant levels. Oxidative stress is an important mechanism underlying the toxic effects of malathion. Malathion-induced neurotoxicity is closely related to oxidative stress caused by malathion. Malathion initially increases cholinergic activity upon acute exposure by inhibiting acetylcholinesterase, which is its primary pathological mechanism. Malathion also induces non-cholinergic neuronal cell death in neurodegenerative conditions. Malathion alters endogenous enzymatic and non-enzymatic antioxidant activities in the brain so, it can lead to mitochondrial malfunction, DNA breakage, apoptosis failure to counteract damage induced by free radicals, including lipid peroxidation [5, 6]. Malathion has been shown to increase malondialdehyde levels, total oxidative levels, and oxidative stress and to impede protective antioxidant machinery, including alterations in TAC and SOD activity [5, 6, 37, 38].

Malathion-induced oxidative stress can result in autophagy, deoxyribonucleic acid fragmentation, and mitochondrial dysfunction, all of which can result in apoptosis. In hippocampus, malathion causes submitochondrial vesicles to produce more reactive oxygen species, which inhibit mitochondrial complexes I, II, and IV. This subsequently alters cellular energy and causes an imbalance between oxidants and antioxidants [39, 40].

Both DNA damage and mitochondrial dysfunction can induce apoptosis. Malathion has been shown to reduce the levels of several antiapoptotic proteins, such as phosphorylated AKT and BCL-2. Malathion induced apoptosis by increasing the amount of Bax/Bcl2 ratio, and caspase 3, in the brain is noteworthy [6, 41, 42].

A number of polyphenols, including flavonoids, anthocyanins, and phenolic acids, have been shown to be effective against malathion-induced tissue damage [43]. Akbel *et al*. [44] demonstrated that resveratrol reduced the amount of malaoxon (breakdown product of malathion) in brain tissues, neutralized the harmful effects of malathion, and preserved the antioxidant defense.

Previous studies [45, 46] reported that supplemental vitamin C significantly reduced neurotoxicity and oxidative stress induced by an insecticide mixture, including malathion. Robea *et al*. [45] reported that compared to the control group, vitamin C-administered group had lower oxidative stress, indicating the protective effect of antioxidants in reducing brain injury. In this study, cotreatment with RGSE, including vitamin C led to a significant reduction in oxidative stress.

## Conclusion

In this study, RGSE and vitamin C provided protection from malathion-induced neurotoxicity in the rat brain by increasing acetylcholinesterase and SOD activity, BDNF level, and TAC and by reducing caspase 3 activity, thereby alleviating apoptosis. Red grape seed extract and vitamin C should be recommended for neuroprotection in individuals exposed to malathion. Further investigation is warranted to clarify the involvement of RGSE and vitamin C in the regulation of acetylcholinesterase and SOD activity, BDNF levels, and TAC.

## Author’s Contributions

MJS: Conceived and designed the study, conducted the study, collected and organized the data, analyzed the data, interpreted the results and wrote the initial and final drafts of the manuscript. The author has read, reviewed, and approved the final manuscript.

## References

[1] Vasseghian Y, Almomani F, Moradi M, Dragoi E.N (2022). Decontamination of toxic Malathion pesticide in aqueous solutions by Fenton-based processes:Degradation pathway, toxicity assessment and health risk assessment. J. Hazard. Mater.

[2] Nascimento M.M, da Rocha G.O, de Andrade J.B (2017). Pesticides in fine airborne particles:From a green analysis method to atmospheric characterization and risk assessment. Sci. Rep.

[3] Baldissera M.D, Souza C.F, Zanella R, Prestes O.D, Meinhart A.D, Da Silva A.S, Baldisserotto B (2021). Behavioral impairment and neurotoxic responses of silver catfish *Rhamdia quelen* exposed to organophosphate pesticide trichlorfon:Protective effects of diet containing rutin. Comp. Biochem. Physiol. C Toxicol. Pharmacol.

[4] Brocardo P.S, Pandolfo P, Takahashi R.N, Rodrigues A.L.S, Dafre A.L (2005). Antioxidant defenses and lipid peroxidation in the cerebral cortex and hippocampus following acute exposure to malathion and/or zinc chloride. Toxicology.

[5] Fortunato J.J, Feier G, Vitali A.M, Petronilho F.C, Dal-Pizzol F, Quevedo J (2006). Malathion-induced oxidative stress in rat brain regions. Neurochem. Res.

[6] Varol S, Başarslan S.K, Fırat U, Alp H, Uzar E, Arıkanoğlu A, Evliyaoğlu O, Acar A, Yücel Y, Kıbrıslı E, Gökalp O (2015). Detection of borderline dosage of malathion intoxication in a rat's brain. Eur. Rev. Med. Pharmacol. Sci.

[7] Dassanayake T.L, Weerasinghe V.S, Gawarammana I, Buckley N.A (2021). Subacute and chronic neuropsychological sequalae of acute organophosphate pesticide self-poisoning:A prospective cohort study from Sri Lanka. Clin Toxicol.

[8] Farnham A, Fuhrimann S, Staudacher P, Quirós-Lépiz M, Hyland C, Winkler M.S, Mora A.M (2021). Long-term neurological and psychological distress symptoms among smallholder farmers in Costa Rica with a history of acute pesticide poisoning. Int. J. Environ. Res.

[9] Saylam C, Üçerler H, Kitiş Ö, Ozand E, Gönül A.S (2006). Reduced hippocampal volume in drug-free depressed patients. Surg. Radiol. Anat.

[10] Bathina S, Das U.N (2015). Brain-derived neurotrophic factor and its clinical implications. Arch. Med. Sci.

[11] Park H, Poo M.M (2013). Neurotrophin regulation of neural circuit development and function. Nat. Rev. Neurosci.

[12] Longo F.M, Massa S.M (2013). Small-molecule modulation of neurotrophin receptors:A strategy for the treatment of neurological disease. Nat. Rev. Drug Discov.

[13] Shi J, Yu J, Pohorly J.E, Kakuda Y (2003). Polyphenolics in grape seeds-biochemistry and functionality. J. Med. Food.

[14] Ben Youssef S, Brisson G, Doucet-Beaupré H, Castonguay A.M, Gora C, Amri M, Lévesque M (2021). Neuroprotective benefits of grape seed and skin extract in a mouse model of Parkinson's disease. Nutr. Neurosci.

[15] Feng Y, Liu Y.M, Fratkins J.D, LeBlanc M.H (2005). Grape seed extract suppresses lipid peroxidation and reduces hypoxic ischemic brain injury in neonatal rats. Brain Res. Bull.

[16] Uysal M, Karaman S (2018). *In vivo* effects of intravenous lipid emulsion on lung tissue in an experimental model of acute malathion intoxication. Toxicol. Ind. Health.

[17] Koracevic D, Koracevic G, Djordjevic V, Andrejevic S, Cosic V (2001). Method for the measurement of antioxidant activity in human fluids. J. Clin. Pathol.

[18] Kovalchuk Y, Hanse E, Kafitz K.W, Konnerth A (2002). Postsynaptic induction of BDNF-mediated long-term potentiation. Science.

[19] Cohen G.M ((1997)). Caspases:The executioners of apoptosis. Biochem. J.

[20] Idrisova K.F, Zeinalova A.K, Masgutova G.A, Bogov A.A, Allegrucci C, Syromiatnikova V.Y, Salafutdinov I.I, Garanina E.E, Andreeva D.I, Kadyrov A.A, Rizvanov A.A, Masgutov R.F (2022). Application of neurotrophic and proangiogenic factors as therapy after peripheral nervous system injury. Neural. Regen. Res.

[21] Dorri S.A, Hosseinzadeh H, Abnous K, Hasani F.V, Robati R.Y, Razavi B.M (2015). Involvement of brain-derived neurotrophic factor (BDNF) on malathion induced depressive-like behavior in subacute exposure and protective effects of crocin. Iran. J. Basic Med. Sci.

[22] Fanaei H, Karimian S.M, Sadeghipour H.R, Hassanzade G, Kasaeian A, Attari F, Khayat S, Ramezani V, Javadimehr M (2014). Testosterone enhances functional recovery after stroke through promotion of antioxidant defenses, BDNF levels and neurogenesis in male rats. Brain Res.

[23] Jaeger B.N, Parylak S.L, Gage F.H (2018). Mechanisms of dietary flavonoid action in neuronal function and neuroinflammation. Mol. Aspects Med.

[24] Sharma P, Kumar A, Singh D (2019). Dietary flavonoids interaction with CREB-BDNF pathway:An unconventional approach for comprehensive management of epilepsy. Curr. Neuropharmacol.

[25] Moosavi F, Hosseini R, Saso L, Firuzi O (2016). Modulation of neurotrophic signaling pathways by polyphenols. Drug Des. Devel. Ther.

[26] Karak P (2019). Biological activities of flavonoids:An overview. Int. J. Pharm. Sci. Res.

[27] Vidyasagar J, Karunakar N, Reddy M.S, Rajnarayana K, Surender T, Krishna D.R (2004). Oxidative stress and antioxidant status in acute organophosphorus insecticide poisoning. Indian J. Pharm.

[28] Meijer M, Hamers T, Westerink R.H.S (2014). Acute disturbance of calcium homeostasis in PC12 cells as a novel mechanism of action for (sub) micromolar concentrations of organophosphate insecticides. Neurotoxicology.

[29] Hargreaves A.J (2012). Neurodegenerations induced by organophosphorus compounds. Adv. Exp. Med. Biol.

[30] Halliwell B (2006). Oxidative stress and neurodegeneration:Where are we now?*J*. Neurochem.

[31] Ozsoy A.Z, Nursal A.F, Karsli M.F, Uysal M, Alici O, Butun I, Tas U, Delibas I.B (2016). Protective effect of intravenous lipid emulsion treatment on malathion-induced ovarian toxicity in female rats. Eur. Rev. Med. Pharmacol. Sci.

[32] Akhgari M, Abdollahi M, Kebryaeezadeh A, Hosseini R, Sabzevari O (2003). Biochemical evidence for free radical-induced lipid peroxidation as a mechanism for subchronic toxicity of malathion in blood and liver of rats. Hum. Exp. Toxicol.

[33] Franco J.L, Posser T, Mattos J.J, Trevisan R, Brocardo P.S, Rodrigues A.L.S, Leal R.B, Farina M, Marques M.R.F, Bainy A.C.D, Dafre A.L (2009). Zinc reverses malathion induced impairment in antioxidant defenses. Toxicol. Lett.

[34] Ullah S, Li Z, Hasan Z, Khan S.U, Fahad S (2018). Malathion induced oxidative stress leads to histopathological and biochemical toxicity in the liver of rohu (*Labeo rohita*, Hamilton) at acute concentration. Ecotoxicol. Environ. Saf.

[35] Thakur S, Dhiman M, Mantha A.K (2018). APE1 modulates cellular responses to organophosphate pesticide-induced oxidative damage in non-small cell lung carcinoma A549 cells. Mol. Cell. Biochem.

[36] Dhouib I.E.B, Lasram M.M, Annabi A, Gharbi N, El-Fazaa S (2015). A comparative study on toxicity induced by carbosulfan and malathion in Wistar rat liver and spleen. Pestic. Biochem. Physiol.

[37] Yu Y, Yang A, Zhang J, Hu S (2013). Maternal exposure to the mixture of organophosphorus pesticides induces reproductive dysfunction in the offspring. Environ. Toxicol.

[38] Mohammadzadeh L, Abnous K, Razavi B.M, Hosseinzadeh H (2020). Crocin-protected malathion-induced spatial memory deficits by inhibiting TAU protein hyperphosphorylation and antiapoptotic effects. Nutr. Neurosci.

[39] Delgado E.H, Streck E.L, Quevedo J.L, Dal-Pizzol F (2006). Mitochondrial respiratory dysfunction and oxidative stress after chronic malathion exposure. Neurochem. Res.

[40] Karami-Mohajeri S, Hadian M.R, Fouladdel S, Azizi E, Ghahramani M.H, Hosseini R, Abdollahi M (2014). Mechanisms of muscular electrophysiological and mitochondrial dysfunction following exposure to malathion, an organophosphorus pesticide. Hum. Exp. Toxicol.

[41] Venkatesan R, Park Y.U, Ji E, Yeo E.J, Kim S.Y (2017). Malathion increases apoptotic cell death by inducing lysosomal membrane permeabilization in N2a neuroblastoma cells:A model for neurodegeneration in Alzheimer's disease. Cell Death Discov.

[42] Salama O.A, Attia M.M, Abdelrazek M.A (2019). Modulatory effects of swimming exercise against malathion induced neurotoxicity in male and female rats. Pestic. Biochem. Physiol.

[43] Madrigal-Santillán E, Madrigal-Bujaidar E, Álvarez-González I, Sumaya-Martínez M.T, Gutiérrez-Salinas J, Bautista M, Morales-González A, García-Luna y González-Rubio M, Aguilar-Faisal I.J, Morales-González J.A (2014). Review of natural products with hepatoprotective effects. World J. Gastroenterol.

[44] Akbel E, Arslan-Acaroz D, Demirel H.H, Kucukkurt I, Ince S (2018). The subchronic exposure to malathion, an organophosphate pesticide, causes lipid peroxidation, oxidative stress, and tissue damage in rats:The protective role of resveratrol. Toxicol. Res. (Camb).

[45] Robea M.A, Jijie R, Nicoara M, Plavan G, Ciobica A.S, Solcan C, Audira G, Hsiao C.D, Strungaru S.A (2020). Vitamin C attenuates oxidative stress and behavioral abnormalities triggered by fipronil and pyriproxyfen insecticide chronic exposure on zebrafish juvenile. Antioxidants.

[46] Elzoghby R.R, Ahlam F.H, Abdel-Fatah A, Farouk M (2014). Protective role of vitamin C and green tea extract on malathion-induced hepatotoxicity and nephrotoxicity in rats. Am. J. Pharmacol. Toxicol.

